# Burden of disease and real-world treatment patterns of patients with systemic lupus erythematosus in the Australian OPAL dataset

**DOI:** 10.1007/s10067-023-06681-x

**Published:** 2023-07-05

**Authors:** Sabina Ciciriello, Geoffrey Littlejohn, Catherine O’Sullivan, Tegan Smith, Claire T. Deakin

**Affiliations:** 1 OPAL Rheumatology Ltd., Sydney, NSW Australia; 2 Royal Melbourne Hospital, Melbourne, VIC Australia; 3 Department of Medicine, Monash University, VIC Clayton, Australia; 4 Centre for Adolescent Rheumatology Versus Arthritis at University College London, University College London Hospitals and Great Ormond Street Hospital, London, UK; 5 National Institute of Health Research Biomedical Centre at Great Ormond Street Hospital, London, UK

**Keywords:** Systemic lupus erythematosus, Lupus, Real-world data, Community care, OPAL

## Abstract

**Objective:**

To describe the demographics, disease burden and real-world management of patients with systemic lupus erythematosus (SLE) in Australian community practice.

**Methods:**

Patients with a physician diagnosis of SLE and at least 1 visit between 1 January 2009 and 31 March 2021 were identified in the OPAL dataset, an aggregated collection of data extracted from the electronic medical records of patients managed by 112 Australian rheumatologists. Demographics, basic clinical features and prescribed medications were described, with medication combinations used as a surrogate of disease severity.

**Results:**

Of 5133 patients with a diagnosis of lupus, 4260 (83%) had SLE. Of these SLE patients, almost 90% of patients were female, with a median age of 49 years [IQR 37–61] at first-recorded visit. Of the 2285 SLE patients whose most recent visit was between 1 January 2019 and 31 March 2021, 52.5% had mild disease, 29.9% had moderate-severe disease and 7.4% had very severe disease. Visit frequency increased with disease severity. Most patients (85.8%) were treated with hydroxychloroquine, typically prescribed as first line-of-therapy.

**Conclusion:**

In this large real-world Australian cohort of patients with SLE, a substantial burden of disease was identified, with a significant proportion (almost one-third of patients) considered to have moderate to severe disease based on medication use. This study provides a greater understanding of the path from symptom onset to treatment and the heterogeneous presentation of patients with SLE who are treated in community practice in Australia.

**Table:** 

**Key messages** *• Most published studies describing patients with SLE are derived from specialist lupus centres, typically in the hospital setting, therefore little is known about the characteristics of patients with SLE who are receiving routine care in community clinics.* *• The OPAL dataset is a large collection of clinical data from the electronic medical records of rheumatologists predominantly practising in private community clinics, which is where the majority (73–80%) of adult rheumatology services are conducted in Australia *[1–3] *. Since data from community care has not been widely available for SLE research, this study contributes important insight into this large and under-reported patient population.* *• To improve access to care and effective treatments, and reduce the burden of SLE in Australia, a greater understanding of the characteristics and unmet needs of patients with SLE managed in the community setting is required.*

**Supplementary Information:**

The online version contains supplementary material available at 10.1007/s10067-023-06681-x.

## Introduction

Systemic lupus erythematosus (SLE) is a complex multisystem, autoimmune disease with diverse clinical manifestations. SLE predominately affects women of childbearing age with symptoms ranging from mild to life-threatening, depending on the severity and pattern of organ system involvement [4]. The clinical manifestations of SLE are notoriously heterogenous and reflect the complex molecular mechanisms underlying the pathogenesis of this disease which involve interactions among genetic, epigenetic, immunological, endocrine and environmental factors [5].

Over the past 50 years, mortality from SLE has significantly improved from a 50% 5-year survival rate in the 1950s [6] to a 95% 5-year survival rate in more recent years [7]. Much of this improvement was brought about by the introduction of corticosteroids and immunosuppressive drugs such as cyclophosphamide (CYC), azathioprine (AZA) and mycophenolate (MPA); however, over the past 35–40 years, the introduction of new therapies for the treatment of SLE has stagnated. Although mortality for patients with SLE has greatly improved, there is still significant morbidity, including long-term organ failure, related to both active disease and toxicity from treatments, particularly corticosteroids [8]. Many patients continue to experience greatly reduced health-related quality of life and persistent fatigue [9]. Furthermore, there remains a subgroup of patients’ refractory to standard treatment regimens, requiring long-term continuation of corticosteroids to maintain disease control, or experimentation with off-label immunomodulating agents. For these patients, safe and effective therapies are urgently needed.

The anti-interferon receptor antibody anifrolumab is the only new therapy in recent years to have successfully received approval from the US Food and Drug Administration (FDA) as well as the Australian Therapeutic Goods administration (TGA) [10]. Despite TGA approval for anifrolumab and belimumab use in Australia, access for patients is limited as these drugs are not reimbursed by the Australian Pharmaceutical Benefit Scheme. This was also the case for rituximab until September 2022. There are several promising agents in phase 2 development, including emapalumab, an interferon gamma (IFNy) inhibitor (NCT05162586), an IL-2 receptor agonist (NCT04433585) and a toll-like receptor (TLR) 7 and TLR8 antagonist (NCT05001737). These new medications hold the potential to provide more targeted and effective treatment options for SLE patients, offering hope for improved disease management and better patient outcomes.

Most published studies describing patients with SLE are derived from populations being treated within specialist lupus centres within hospitals. In Australia, approximately 73–80% of routine rheumatology care is delivered in private community-based clinics [1–3] whose data are typically unavailable for observational research. As such, little is known about the characteristics of SLE patients attending community-based clinics, or the approach to disease management delivered by the majority of Australian rheumatologists. Furthermore, the accepted disease activity measures for SLE, including the Systemic Lupus Erythematosus Disease Activity Index (SLEDAI), British Isles Lupus Activity Group (BILAG), Systemic Lupus Activity Measure (SLAM), can be time-consuming and are unlikely to be used routinely during clinical management [11]. Consequently, these disease activity measures are not typically available for patients treated in routine care in Australia, and other approaches to understanding the burden of SLE are needed.

OPAL Rheumatology is a consortium of rheumatologists using a bespoke electronic medical record (EMR) in their routine clinical practice. Deidentified clinical data captured at the point-of-care are extracted from all participating sites on a quarterly basis and aggregated to create the OPAL dataset [12]. At present 112 rheumatologists (approximately one third of Australian rheumatologists) practising in 43 predominantly private community-based clinics around Australia are contributing their clinical records to this initiative. These clinics are located in the states of Queensland, New South Wales, Victoria, Western Australia, Tasmania and the Australian Capital Territory, with a dominance for Victoria and New South Wales which reflects the distribution of the Australian population.

This non-interventional, observational study aimed to use the real-world OPAL dataset to describe the demographics, burden of disease and treatment patterns among Australian patients with SLE being treated in the community setting. Given the growing interest in EMR as a source of real-world data, this study is also a useful exemplar of how to extract and infer meaningful information from EMR to understand a population of patients with SLE who may be under-represented in research [13].

## Patients and methods

### Data source

This was a retrospective, non-interventional cohort study of patients diagnosed with SLE in the Australian OPAL dataset. Clinical information captured during the routine consultation was entered into the patient’s EMR (Audit4, Software4Specialists Pty Ltd, Australia) by the clinician, and pathology reports were electronically transferred from pathology providers and deposited in the EMR. Audit4 serves as the clinician’s prescribing software and a record of medications is maintained within the patient’s EMR.

EMR data better reflects the diversity of patients in real-world healthcare settings compared with clinical trials and utilising EMR data is particularly useful for providing valuable insights into the real-life management of disorders such as SLE, in everyday clinical practice. No data fields are compulsory for clinicians that contribute to the OPAL dataset, and the treating rheumatologist enters the information they require to accurately capture the patient’s disease state as well as the data needed to effectively manage the patient long-term. Data available for this study included patient demographics (age, sex, living/deceased), disease history (symptom onset as provided by the patient or primary care physician, visit history), clinical features, inflammatory markers (erythrocyte sedimentation rate (ESR), C-reactive protein (CRP)), pathology measures indicative of cytopaenias, and medication history. Unfortunately, at the time of this study, the EMR did not contain dedicated fields for structured collection of disease activity measures such as the SLEDAI and BILAG so disease activity could not be reported. Additional limitations of utilising EMR data are included in the limitations section. Data were deidentified to patient, clinician and clinic prior to extraction from the clinician’s server and aggregated across all sites prior to analysis.

The activities of OPAL Rheumatology Ltd have received overarching ethics approval from the University of New South Wales (UNSW) Human Research Ethics Committee (HREC), based on a patient opt-out arrangement (HC17799). This research protocol was approved by the UNSW HREC (HC210163).

### Patient population and eligibility criteria

Patients from the OPAL dataset were included in the study if they were diagnosed with SLE according to the following ICD10 codes (M32.10, M32.11, M32.12, M32.13, M32.14, M32.15, M32.19, M32.8, M32.9, N29.8), aged at least 18 years of age and had at least one visit date recorded within the study window (1 January 2009–31 March 2021). For analyses of the most recent visit, only patients that were recorded as having a visit between 1 January 2019 and 31 March 2021 were included. This more recent timeframe was analysed to gain a more current understanding of the demographics and management of patients with SLE in the OPAL dataset. Patients were excluded if they had no visit data recorded. We also excluded other forms of lupus including cutaneous lupus, drug induced SLE and positive lupus anti-coagulant (L93.0, L93.1, L93.2, M32.0, R76.8). For further details on the ICD10 codes please see Supplementary Table 1.

### Disease severity stratification by treatment

**Fig. 1:**
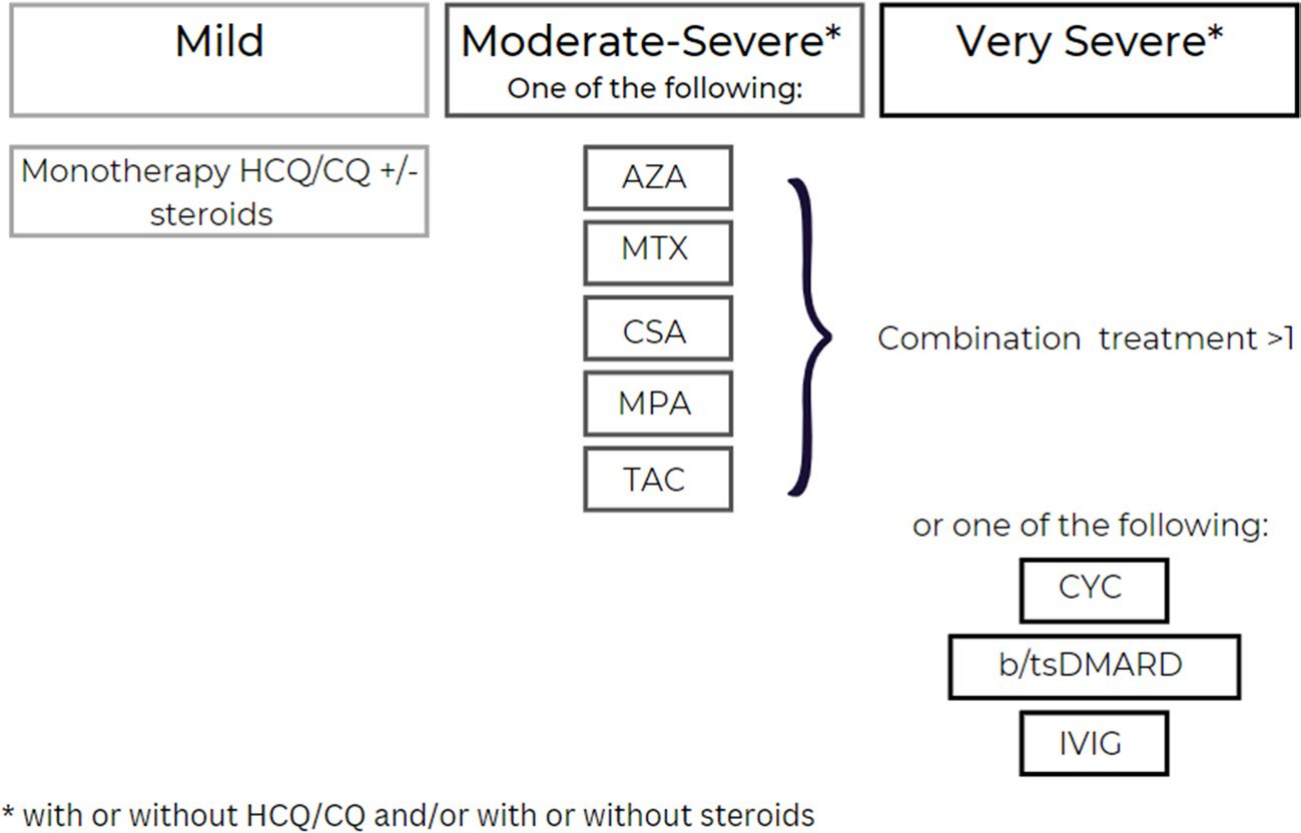
Disease severity stratification by treatment. Mild disease: treatment with monotherapy HCQ or CQ, with or without corticosteroids. Moderate to severe disease: treatment with one of the following; AZA, MTX, CSA, MPA or TAC, with or without HCQ, CQ or corticosteroid. Severe disease: combination therapy with > 1 of the following agents: AZA, MTX, CSA, MPA or TAC or treatment with one of the following; CYC, any b/tsDMARD or IVIG, with or without HCQ or CQ or corticosteroids. HCQ/CQ; hydroxychloroquine/ chloroquine, AZA; azathioprine, MTX; methotrexate, CSA; cyclosporin, MPA; mycophenolate, TAC; tacrolimus, CYC; cyclophosphamide, b/tsDMARD; biologic or targeted synthetic disease-modifying anti-rheumatic drug (b/tsDMARD), IVIG; intravenous immunoglobulin

Since the OPAL dataset does not currently capture the clinical manifestations of SLE in a uniform and detailed manner to generate validated disease activity measures, a surrogate measure of disease severity was developed as follows. The 2019 update of the European Alliance of Associations for Rheumatology (EULAR) recommendations for the management of SLE summarises the combinations of drugs used according to disease severity stratification [14]. We utilised a similar method based on this stratification to assign patients to a disease severity rank of either (i) mild, (ii) moderate–severe and (iii) very severe according to their recorded medication history. Treatment of a patient with (i) an anti-malarial (hydroxychloroquine (HCQ) or chloroquine (CQ)) as monotherapy, with or without corticosteroids, was designated mild disease, (ii) treatment with one of the following agents: AZA, methotrexate (MTX), cyclosporin (CSA), MPA or tacrolimus (TAC), with or without HCQ, CQ or corticosteroids was used as a surrogate of moderate–severe disease and (iii) treatment with cyclophosphamide (CYC), any biologic or targeted synthetic disease-modifying anti-rheumatic drug (b/tsDMARD), or intravenous immunoglobulin (IVIG); or combination therapy with more than one of the following agents: AZA, methotrexate (MTX), cyclosporin (CSA), MPA or tacrolimus (TAC), with or without HCQ or CQ or corticosteroids were designated very severe disease (Fig. 1). Patients on steroids alone (monotherapy or combinations of steroids) were also described. It is noted that medication use fluctuates over time according to disease activity, particularly during flares. We have selected those that were used to treat the maximal disease activity during the observation time. Where patients had multiple combinations of medications that satisfied different definitions of disease activity, the most severe definition was selected.

### Statistical methods

This study used descriptive statistics for analyses of all endpoints. Numeric variables were described using median, interquartile range (IQR) and range; any variables that were approximately normally distributed were summarised using mean and standard deviation (SD). Categorical and binary variables were described using counts (*n*) and percentages. Percentages of missing data are described. Where percentages were calculated for a given variable, these were the total for that variable out of the total number of patients with data available for that variable to avoid underestimation due to missing data. All analyses were performed using R version 4.0.2.

### Patient and public involvement statement

Patients were not directly involved in the design or conduct of this study.

## Results

### Lupus-related diagnoses

A large population (*N* = 5133) of eligible patients with SLE or other forms of lupus and at least one visit after 1 January 2009 were identified in the OPAL dataset. These patients included 4260 patients with SLE (83%) which was mostly comprised of “SLE unspecified” (94.2%), followed by lupus nephritis (8.2%) (Supplementary Table 1). Of these patients, 2636 patients had their most recent visit after 1 January 2019 and were considered to represent a “recent snapshot” of the SLE population. The remaining 873 patients with other forms of lupus (17%) were predominantly diagnosed with cutaneous lupus. This study focussed on the patients diagnosed with SLE.

### Demographics, disease history and visit frequency

The demographics, disease history and frequency of visits of patients with SLE are reported in Table 1. As expected, the majority of patients were female (89.8%). The median age at first recorded visit was 49 years [IQR 37–61] and age at most-recent visit was 53 years [IQR 40–66], reflecting length of follow-up. The median duration of disease recorded was 2.3 years [IQR 0.4–6.1] and the time from symptom onset to first recorded visit was 8.5 years [IQR 2.2–17.0]. The median number of visits recorded was 7 visits [3–16] with a median interval between visits of 3 months [2–6] (Table 1).

**Table 1:** Demographics, disease history and frequency of visits of patients with SLE

Feature	Category	Total (*n* = 4260)
Gender, *n* (%)	Female	3779 (89.8%)
	Male	429 (10.2%)
Age at first-recorded visit (years), median [IQR]	–	49 [37–61]
Age category at first-recorded visit, *n* (%)	18–30 years	525 (12.3%)
	31–40 years	830 (19.5%)
	41–50 years	862 (20.2%)
	51–60 years	865 (20.3%)
	61–70 years	724 (17%)
	71–80 years	327 (7.7%)
	81 + years	88 (2.1%)
Age at most-recent visit (years), median [IQR]	–	53 [40–66]
Age category at most-recent visit, *n* (%)	18–30 years	382 (9%)
	31–40 years	698 (16.4%)
	41–50 years	836 (19.6%)
	51–60 years	857 (20.1%)
	61–70 years	788 (18.5%)
	71–80 years	514 (12.1%)
	81 + years	172 (4%)
Duration of disease recorded in Audit4 (years), median [IQR]	–	2.3 [0.4–6.1]
Recorded disease duration category, *n* (%)	0–2.0 years	2007 (47.1%)
	2.1 – 5.0 years	935 (21.9%)
	5.1 – 10.0 years	962 (22.6%)
	Over 10 years	356 (8.4%)
Time from symptom onset to first recorded visit, median [IQR]	–	8.5 [2.2 – 17.0]
Number of recorded visits*, median [IQR]	–	7 [3–16]
Frequency of visits per year, median [IQR]	–	2.9 [1.9–4.3]
Visit frequency category, *n* (%)	Less than 2 visits	754 (27.2%)
	2–5 visits inclusive	1544 (55.8%)
	Over 5 visits	471 (17%)
Interval between visits (months)*, median [IQR]	–	3 [2–6]
Visit interval category, *n* (%)	Less than 3 months	1174 (39.6%)
	3–6 months inclusive	1432 (48.3%)
	Over 6 months	360 (12.1%)
Pathology at most recent visit, median [IQR]	ESR (mm/h)	10 [5–23]
	CRP (mg/L)	3.5 [1–5]
	Leukocytes (× 10^9^/L)	5.9 [4.6–7.5]
	Platelets (× 10^9^/L)	253 [210–300]
	Neutrophils (× 10^9^/L)	3.6 [2.5–4.85]

^*^To exclude patients whose frequencies of visits may appear to be inflated due to limited follow-up, the frequencies were described for patients with at least 1 year of follow-up only (*n* = 3202). Median interval between visits was reported for patients with at least 4 recorded visits (*n* = 3425)

When disease duration was cross-tabulated by demographic features for patients with SLE the median time from symptom onset to first recorded visit to a clinician contributing to the OPAL dataset was found to be longer for females than males at 9 years [2.7–17.5] compared to 3.7 years [1–12.9], respectively, although these data should be interpreted cautiously due to a high level of missing data for date of disease onset, and may also reflect some time spent in care elsewhere prior to being seen by the OPAL rheumatologist. Younger patients (< 40) had slightly more frequent visits as well as a shorter interval between visits (Table 2).

**Table 2:** Cross-tabulation of disease duration against demographic groups for patients with SLE

Demographic group	Category	Duration of disease recorded in Audit4 (years)	Time from symptom onset to first-recorded visit (years)	Number of visits per year	Median interval between visits per patient (months)
SLE (*n* = 4260), median [IQR]					
Gender	Female	2.4 [0.4–6.2]	9 [2.7–17.5]	2.9 [1.9–4.3]	3 [2–6]
	Male	2.2 [0.3–5.9]	4.4 [1.0–12.7]	2.9 [1.9–4.5]	3.0 [2.0–6.0]
Age at most–recent visit	18–30 years	0.8 [0.1–2.6]	3 [0.9–7.0]	3.3 [2.3–5.0]	2.0 [1.0–4.0]
	31–40 years	1.3 [0.2–4.0]	4.8 [1.0–10.0]	3.5 [2.1–5.0]	2.0 [1.0–4.0]
	41–50 years	2.2 [0.3–6.1]	7.4 [2.1–15.0]	2.9 [1.8–4.6]	3.0 [2.0–5.0]
	51–60 years	3.0 [0.5–6.5]	10.5 [3.4–19.7]	2.7 [1.8–3.8]	4.0 [2.0–6.0]
	61–70 years	3.0 [0.6–7.1]	11.4 [4.0–2.2]	2.9 [1.8–4.2]	4.0 [2.0–6.0]
	71–80 years	4.4 [1.3–7.7]	14.2 [6.6–24.3]	2.5 [1.8–3.8]	4.0 [2.0–6.0]
	81 + years	3.5 [0.6–7.6]	10.2 [4.3–18.4]	2.6 [1.9–4.0]	4.0 [2.0–6.0]

### Medication utilisation

Medication data was recorded for 81.8% of patients with SLE (*n* = 3484/4260). The total number of different immunomodulatory medications received by patients during their recorded disease history are summarised in Supplementary Table 2. In brief, the majority of patients (68.2%) had less than 3 different medications recorded, 31.8% had 3–5 medications recorded and 1.8% had more than 5 medications recorded during their disease history. The total number of different medications prescribed increased with increasing duration of disease (Supplementary Table 2).

Details of medications of interest received by patients with SLE are described in Table 3. The majority of patients (85.8%) had received HCQ or CQ at some point in their recorded disease history and this was typically prescribed as the patients’ first line of therapy. Corticosteroids, MTX and AZA were prescribed in 65.8%, 28.3% and 13% of patients, respectively, and these medications were most commonly used as second line treatment. MPA (11.5%), CYC (0.4%), CSA (0.5%), TAC (0.5%), RTX (0.9%), BEL (0%) and other b/tsDMARDs (4.7%) were used less frequently and typically given in later lines (third line onwards) of therapy (Table 3).

**Table 3:** Medications received by patients with SLE

Medication	Number of patients, *n* (%)	Initial prescribed dose (mg/day), median [IQR]	Duration of treatment (months), median [IQR]	Line of treatment, median [IQR]	Time from first- recorded visit to treatment start (months), median [IQR]
Steroids PDN	855 (24.5%)	5 [5–9]	7 [6–32.65]	2 [1, 2]	2 [0–18]
PRDL	1439 (41.3%)	5 [4–10]	12 [6 – 38.35]	2 [1, 2]	0 [0–13]
DEX	4 (0.1%)	–	4.75 [3.025–8.8]	1.5 [1–2.5]	7.5 [1.5–13.25]
HCQ or CQ	2989 (85.8%)	400 [200–400]	21.4 [6–71]	1 [1, 2]	0 [0–2]
MTX	986 (28.3%)	2.1 [1.4–2.9]	15.85 [6–45.6]	2 [1, 3]	5 [0–25.5]
AZA	454 (13%)	100 [50–100]	15.1 [6–46.9]	2 [1, 3]	4 [0–19]
MPA	400 (11.5%)	1000 [500–1500]	12 [6–32.95]	3 [2, 4]	17 [1 –52]
CYC	15 (0.4%)	62.5 [35.7–100]	6 [4.75–6.5]	3 [2, 4]	13 [1.5–28.5]
CSA	18 (0.5%)	150 [50–200]	6 [5.05 – 10.5]	2 [1.25–5.5]	19 [0–60]
TAC	17 (0.5%)	3 [2, 4]	8.1 [6 – 17.2]	5 [4-6]	48 [13 – 88]
IVIG	1 (0%)	–	6 [6-6]	3 [3–3]	0 [0 – 0]
RTX	32 (0.9%)	71.4 [5.5–71.4]	11.2 [6.2–25.2]	6 [4 – 7.25]	41 [20.3 – 67.8]
BEL	0 (0%)	–	–	–	–
Other b/tsDMARD	164 (4.7%)	10 [4–17.9]	12 [6–26.8]	4 [3–5]	35.5 [8–64.3]

*PRDL* prednisolone, *PDN* prednisone, *HCQ* hydroxychloroquine, *CQ* chloroquine, *AZA* azathioprine, *MTX* methotrexate, *CSA* cyclosporin, *CYC* cyclophosphamide, *TAC* tacrolimus, *MPA* mycophenolate, *BEL* belimumab, *RTX* rituximab, *b/tsDMARD* biologic or targeted synthetic disease-modifying anti-rheumatic drug, *IVIG* intravenous immunoglobulin. ^*^Number of patients is the number of patients who received that drug ever in their recorded disease history. Where a patient received a given drug more than once in their recorded disease history, the details of the first pharmacological intervention was described

### Medication-derived disease severity & visit frequency

In order to explore the burden of disease in this SLE population, medication use was used as a surrogate measure to stratify patients into disease severity categories, similar to those described in the 2019 update of the EULAR recommendations [14].

**Fig. 2:**
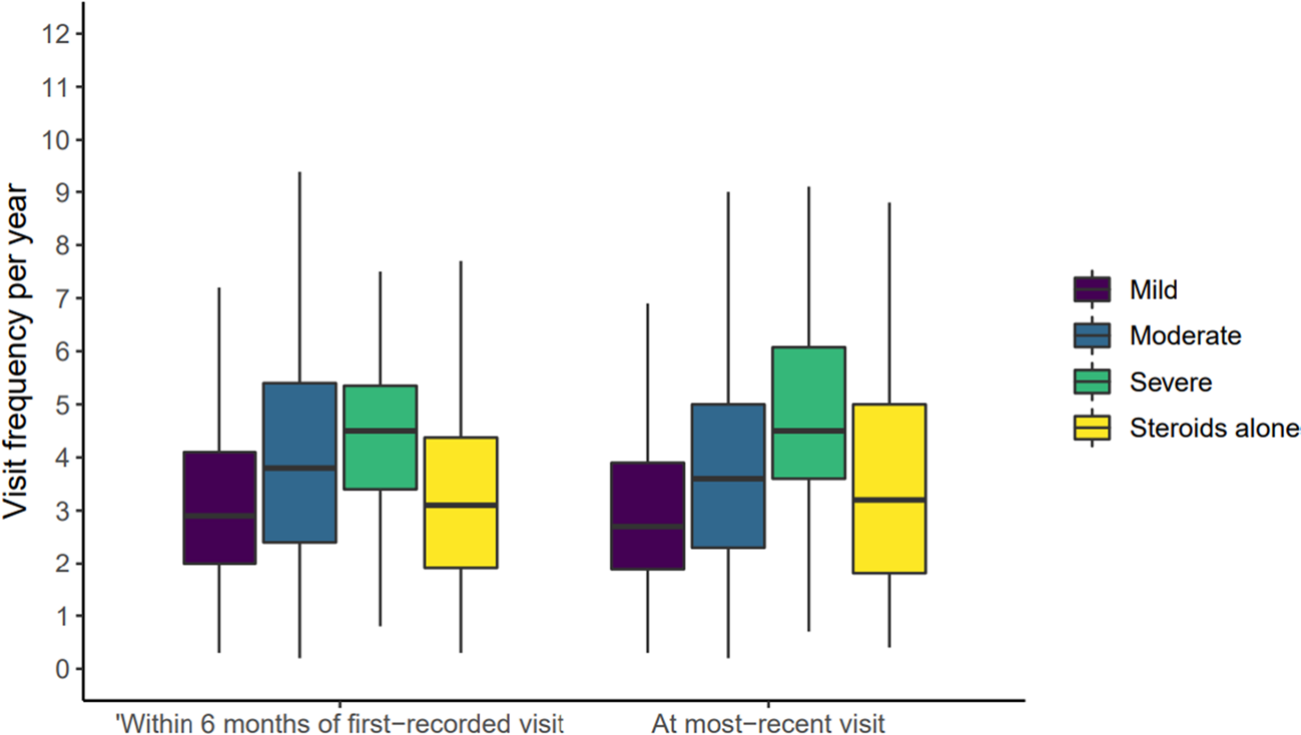
The relationship between visit frequency and disease severity groupings within 6 months of the first-recorded visit and at most recent visit. Median visit frequency per year increased as disease severity increased. ^*^Patients with less than 1 year of follow-up were excluded from the calculation of visit frequency to avoid over-estimation of visit frequency in those patients

Based on this stratification the proportion of patients with “mild,” “moderate–severe,” or “very severe” disease within 6 months of their first-recorded visit, as well as at their most recent visit, are summarised in Table 4. During the first 6 months of recorded disease history after the first recorded visit, 61% of patients were treated in a manner that would reflect “mild” disease severity, 29.3% of patients were treated in a manner that would reflect “moderate-severe” disease and 2.6% were treated with medications associated with “very severe” disease. At the most recent visit, there was a slightly smaller proportion of patients in the “mild” group (52.5%) and a slightly higher proportion of patients in the “very severe” group (7.4%). The proportion of patients in the “moderate-severe” group was similar at the most recent visit. Cross-tabulation of the visit frequency with the medication-derived disease severity categories indicated that the median visit frequency per year increased as disease severity increased. Within six months of first recorded visit, the median [IQR] visit frequency was 2.9 [2–4.2], 3.8 [2.4–5.6], 4.6 [3.4–6.0] and 3.1 [1.9–4.3], for mild, moderate to severe, very severe, and steroid treatment alone, respectively. At most recent visit, the median [IQR] visit frequency was 2.7 [1.9–4.0] for “mild,” 3.7 [2.3–5.0] for moderate to severe, 4.7 [3.6–6.3] for very severe and 3.2 [2.0–5.0] for steroid treatment alone (Fig. 2).

**Table 4:** Patients with mild, moderate to severe or severe disease within 6 months of first–recorded visit and at most recent visit, based on prescribed medication combinations

Surrogate disease severity	Treatment combinations	Within 6 months of first–recorded visit (*n* = 3044)	At most–recent visit (*n* = 2285)
		*n* (%)	*n* (%)
Mild	HCQ or CQ as monotherapy	1857 (61%)	1199 (52.5%)
Moderate–severe	AZA, MTX, CSA, TAC or MPA as monotherapy, regardless of HCQ/CQ or steroids	891 (29.3%)	683 (29.9%)
Very severe	Any use of CYC, any b/tsDMARD or IVIG; or triple-therapy of AZA, MTX, CSA, TAC or MPA	79 (2.6%)	168 (7.4%)
Steroids alone	PRDL/PDN	217 (7.1%)	235 (10.3%)

*PRDL* prednisolone, *PDN* prednisone, *HCQ* hydroxychloroquine, *CQ* chloroquine, *AZA* azathioprine, *MTX* methotrexate, *CSA* cyclosporin, *CYC* cyclophosphamide, *TAC* tacrolimus, *MPA* mycophenolate, *b/tsDMARD* biologic or targeted synthetic disease-modifying anti-rheumatic drug, *IVIG* intravenous immunoglobulin

### Safety

Of 1226 patients with SLE that had a reason for ceasing an immunosuppressant medication recorded by the clinician, 356 (29%) were due to an adverse event. The list of recorded adverse events of interest for each drug (HCQ/QC, MTX, AZA, MPA, CYC, CSA, TAC or IVIG) are summarised in Supplementary Table 3.

The number of recorded retinopathy cases in this population were also investigated. A total of 40 cases (0.9%) were recorded, and this included 18 patients where retinopathy was recorded as the primary reason for discontinuing the medication.

## Discussion

This study represents the largest cohort of patients with SLE managed by rheumatologists in the community setting in Australia. It is recognised that SLE is a heterogeneous disease in its epidemiology, etiology and clinical phenotype. The patients with SLE captured within the OPAL dataset were also found to be a diverse population with the majority of patients currently appearing to have a disease phenotype that requires treatment with medications recommended for mild disease. However, there were also a significant proportion of patients who had required treatment with immunosuppressive or biologic therapies, suggesting more severe disease. Consequently, these data are a valuable complement to data from major specialist centres for understanding the overall burden of SLE in Australia. Since the OPAL dataset is based on an EMR developed for routine clinical management rather than research, this study is also important for establishing which information can be gleaned or inferred from the EMR to better understand this patient group who may not otherwise be well-represented in research. This approach is consistent with a recent study using administrative data to understand the burden of SLE in the UK [13].

The number of patients with lupus nephritis or renal involvement recorded as a concomitant condition was lower in this cohort of patients than would be expected. Renal involvement is one of the most common systemic complications of SLE and has been reported elsewhere to affect approximately 40% of patients with SLE during their lifetime [15, 16]. In this cohort of patients, lupus nephritis was recorded in 8.2% of patients. This may be due to clinicians recording their diagnosis of SLE, but not recording all of the clinical manifestations of the condition. It may also reflect the milder phenotype of SLE being treated in community clinics. The true prevalence of renal involvement in this population may be difficult to ascertain in the absence of a comprehensive chart review and access to data from other specialties such as nephrologists and hospitalisation visits, which would be impractical for this cohort.

Currently diagnosis of SLE is based on the clinical acumen of the treating rheumatologist, supported by laboratory investigation [17]. The heterogeneity and non-specific nature of many manifestations of SLE can impede the early recognition and referral of suspected SLE cases to a rheumatologist which, in turn, can lead to a delay in the patient receiving a diagnosis and commencing appropriate treatment [18]. In this patient population, the median time from symptom onset to first recorded visit with an OPAL rheumatologist was 8.5 years and this was longer for females (9 years) than males (3.7 years). It is important to note that it is not known if the first visit recorded in the OPAL dataset was the patient’s first encounter with a rheumatologist for their SLE symptoms. It is possible that a patient had previously seen another specialist first or changed rheumatologists during their treatment journey. This study only included data that was derived from the EMR of clinicians contributing to OPAL. While some of this may reflect prior care received elsewhere, this observation is consistent with long delays between the onset of first symptoms and the diagnosis of SLE have been reported elsewhere. In a survey of US patients with SLE (*n* = 827), respondents reported an average delay of 2.1 years (SD 4.9) before seeking medical attention from the time they first experienced symptoms and a further 3.5 years (SD 5.4) delay before receiving a formal diagnosis [19]. A cross-sectional analysis of the German LuLa cohort reported a mean duration from symptom onset to diagnosis of 47 months (SD 73) with longer time to diagnosis associated with worse outcomes [18]. Furthermore, a shorter delay in diagnosis of male patients has been reported previously [20, 21].

A recent analysis from the Asia Pacific Lupus Collaboration (APLC) reported on the treatment patterns of a large (*n* = 2860), international, multicentre cohort of patients with SLE [22]. Patients were recruited from 14 hospital-based sites. Some similarities in medication utilisation were observed between the current community-based cohort of Australian patients characterised in this study and the hospital-based APLC cohort. We found that > 85% of patients had ever received an anti-malarial (HCQ or CQ), 54.2% an immunosuppressant (MTX, AZA, MPA, CSA, TAC, CYC) and 5.6% a b/tsDMARD medication. In the APLC cohort, 75% of patients had been treated with an anti-malarial, 68.9% with an immunosuppressant and 2.9% with a b/tsDMARD [22].

Treatment with a corticosteroid plus an anti-malarial plus an immunosuppressant was the most common treatment strategy (31.4% of visits) observed in the APLC cohort study [22]. In the OPAL cohort study presented here, patients treated with an immunosuppressant (AZA, MTX, CSA or TAC) regardless of anti-malarial and steroid (PRDL or PDN) treatment were categorised as having moderate–severe disease activity and accounted for almost a third of the patients. Of note, utilisation of b/tsDMARD therapies in both the APLC and OPAL cohorts was minimal. This is not surprising, however, as there were no b/tsDMARD therapies reimbursed for the treatment of SLE in Australia, nor in most of the regions contributing data to the APLC, at the time of these studies.

Although steroids are the mainstay of therapy for SLE and can be effective, it is well established that they are a major contributor to damage accrual and patient morbidity [8]. In the current study, the majority of patients (65.8%) had received treatment with a corticosteroid. Furthermore, a small but not insignificant proportion of patients (10.3%) were also found to be treated with steroid monotherapy at their most recent visit. The median dose of steroids in this monotherapy treatment group cannot be accurately gathered from the EMR at this time. Adverse side effects of steroids are generally dependent on the dose as well as duration of use but commonly include acne vulgaris, weight gain, metabolic syndrome, diabetes mellitus and hypertension [23]. These may be improved by tapering the dose; however, severe complications such as osteoporosis, cataracts, avascular necrosis (AVN) and cardiovascular events often present later and result in irreversible damage [24]. The high reliance on steroids to manage SLE in the community setting highlights the need for safer and more effective targeted therapies.

The total number of different medications prescribed increased with disease duration. Patients with more severe disease would also be expected to be seen more frequently by their rheumatologist. In agreement with this, visit frequency was found to increase with the disease severity level, indicating that the treatment categories used to classify patients were a reasonable approximation of disease severity in this population.

To provide context to the cohort described in this study in relation to previously published studies, it is important to recognise that differences in demographics, medication utilisation and disease severity across patient cohorts may reflect the setting from which that data was collected (tertiary centre/hospital or community clinic), whether eligibility for inclusion in the study was based on physician diagnosis or meeting classification criteria, whether data was captured for the primary purpose of routine care or research, and the level of consent and involvement required by the patient and clinician to contribute data. This cohort study was based on data captured at the point of care for the primary purpose of routine clinical care, eligibility was based on physician diagnosis of SLE and other forms of lupus, and no other classification criteria was applied. Extraction of deidentified data from the EMR was automated and clinical records were included unless the patient elected to opt out which is reportedly a rare occurrence. Approximately 1 in 3 Australian rheumatologists contributed their clinical records for this study, and the majority of patients were managed in the private community setting, which is representative of the practise of rheumatology in Australia, where 80% of rheumatology care is delivered in private community clinics [1]. Taken together, this cohort study likely represents a significant proportion of patients currently being treated for SLE in Australia.

## Conclusion

Although most patients with SLE in this real-world Australian cohort treated in the community setting were considered to have milder disease, there was a significant proportion (almost one-third of patients) considered to have moderate to severe disease based on medication use. Disease severity was also linked to an increased frequency of rheumatologist visits suggesting that these patients may be sub-optimally controlled with the current available treatments, which puts upwards pressure on the personal, social and economic consequences of SLE. A greater understanding of the path from symptom onset to treatment, the heterogeneous presentation of this disease and predictors of progression to severe disease will be important for improving access to specialty care and ensuring early intervention with effective and safe treatment strategies to reduce the burden of SLE in the community.

## Limitations

This was a retrospective, observational study of a dataset comprising clinical data captured for the primary purpose of routine clinical care. As a result, missing data for various outcome variables was observed. Pathology results that were not returned from the provider in a machine-readable format were unable to be extracted from the EMR for use in this study which may result in an underestimate of the use of pathology measures in the management of SLE, potentially introducing bias. Therefore, markers for monitoring SLE or renal disease were not reported in this study. As mentioned in the methods, at the time of this study, the EMR did not contain dedicated fields for structured collection of disease activity measures such as the SLEDAI and BILAG so disease activity could not be reported. The need, benefit and risk of escalating or de-escalating therapy was at the clinician’s discretion, and clinical management strategies may vary between clinicians which we were unable to control for in this study. Furthermore, increased visits to healthcare providers may provide more opportunities for receiving treatment, and this can be considered a potential confounding factor when using medication as a proxy for disease severity. Some of the medications used in the treatment of SLE can also be used for other conditions. The prevalence of lupus nephritis was likely underreported in this cohort. This may reflect shared care arrangements with nephrologists, and documentation of the diagnosis and management of lupus nephritis being housed in communications between clinicians that were not in a machine-readable format. Results must therefore be interpreted in this light.

### Supplementary Information

Below is the link to the electronic supplementary material.

Supplementary file1 (DOCX 34 KB)

## Data Availability

Data collection is based on opt-out patient consent, and patients have consented to their data being made available to OPAL Rheumatology. Requests for access to summary statistics will be considered by the OPAL Scientific Review Committee.

## References

[1] Arthritis Australia (2017) Australian Healthcare and Hospitals Association Rheumatology Nurses: Adding Value to Arthritis Care

[2] Australian Rheumatology Association (2022) Australian Rheumatology Association 2022 Annual Achievement Report. https://rheumatology.org.au/About/Our-Annual-Achievement-Report. Accessed 22 Oct 2022

[3] Health Workforce Data. https://hwd.health.gov.au/. Accessed 18 Jan 2023

[4] Mok CC (2017). Biological and targeted therapies of systemic lupus erythematosus: evidence and the state of the art. Expert Rev Clin Immunol.

[5] Moulton VR, Suarez-Fueyo A, Meidan E (2017). Pathogenesis of human systemic lupus erythematosus: a cellular perspective. Trends Mol Med.

[6] Merrell M, Shulman LE (1955). Determination of prognosis in chronic disease, illustrated by systemic lupus erythematosus. J Chronic Dis.

[7] Borchers AT, Keen CL, Shoenfeld Y, Gershwin ME (2004). Surviving the butterfly and the wolf: mortality trends in systemic lupus erythematosus. Autoimmun Rev.

[8] Bruce IN, Keeffe AG, Farewell V (2015). Factors associated with damage accrual in patients with systemic lupus erythematosus: results from the Systemic Lupus International Collaborating Clinics (SLICC) Inception Cohort. Ann Rheum Dis.

[9] Bakshi J, Segura BT, Wincup C, Rahman A (2018) Unmet needs in the pathogenesis and treatment of systemic lupus erythematosus. Clin Rev Allergy Immunol 55(3):352–367. 10.1007/s12016-017-8640-5 10.1007/s12016-017-8640-5 PMC6244922 28853005

[10] Burki TK (2021). FDA approval for anifrolumab in patients with lupus. Lancet Rheumatol.

[11] Mikdashi J, Nived O (2015). Measuring disease activity in adults with systemic lupus erythematosus: the challenges of administrative burden and responsiveness to patient concerns in clinical research. Arthritis Res Ther.

[12] Littlejohn GO, Tymms KE, Smith T, Griffiths HT (2020). Using big data from real-world Australian rheumatology encounters to enhance clinical care and research. Clin Exp Rheumatol.

[13] Langham J, Barut V, Samnaliev M (2021). Disease severity, flares and treatment patterns in adults with systemic lupus erythematosus in the UK: a real-world observational retrospective cohort analysis. Rheumatol Adv Pract.

[14] Fanouriakis A, Kostopoulou M, Alunno A (2019). 2019 update of the EULAR recommendations for the management of systemic lupus erythematosus. Ann Rheum Dis.

[15] Ahn SS, Yoo J, Jung SM (2020). Comparison of clinical features and outcomes between patients with early and delayed lupus nephritis. BMC Nephrol.

[16] Davidson A (2016). What is damaging the kidney in lupus nephritis?. Nat Rev Rheumatol.

[17] Bertsias GK, Pamfil C, Fanouriakis A, Boumpas DT (2013). Diagnostic criteria for systemic lupus erythematosus: has the time come?. Nat Rev Rheumatol.

[18] Kernder A, Richter JG, Fischer-Betz R (2021). Delayed diagnosis adversely affects outcome in systemic lupus erythematosus: cross sectional analysis of the LuLa cohort. Lupus.

[19] Al Sawah S, Daly R, Foster S (2015). understanding delay in diagnosis, access to care and satisfaction with care in lupus: findings from a cross-sectional online survey in the United States. Ann Rheum Dis.

[20] Gozcu E, Karatas A, Oz B, Koca SS (2018). PS6:125 Systemic lupus erythematosus diagnosis is earlier in males compared to females. Lupus Sci Med.

[21] Molina JF, Drenkard C, Molina J (1996). Systemic lupus erythematosus in males. a study of 107 Latin American patients. Medicine.

[22] Kandane-Rathnayake R, Louthrenoo W, Luo S-F, et al (2021) Patterns of medication use in systemic lupus erythematosus – a multi-centre cohort study. Arthritis Care Res (Hoboken) n/a. 10.1002/acr.24740 10.1002/acr.24740 34197023

[23] Mejía-Vilet JM, Ayoub I (2021). The use of glucocorticoids in lupus nephritis: new pathways for an old drug. Front Med (Lausanne).

[24] Zonana-Nacach A, Barr SG, Magder LS, Petri M (2000). Damage in systemic lupus erythematosus and its association with corticosteroids. Arthritis Rheum.

