# Supplementary material for: Burden of disease and real-world treatment patterns of patients with systemic lupus erythematosus in the Australian OPAL dataset
Source: Clin Rheumatol. 2023 Jul 5;42(11):2971–80. doi: 10.1007/s10067-023-06681-x (PMC10587330; doi:10.1007/s10067-023-06681-x)
Supplement: Supplementary file 1 — Supplementary file1 (DOCX 34 KB) [file 10067_2023_6681_MOESM1_ESM.docx]

**Supplementary Tables**

**Supplementary Table 1: ICD10 codes comprising lupus patient groups**

| ICD10 Code | Description | SLE (n=4260, 83%) | Other lupus (n=873, 17%) |
| --- | --- | --- | --- |
| M32.1 | SLE with renal involvement/organ or system involvement | 216 (5.1%) | 0 (0%) |
| M32.8 | Other forms of SLE | 10 (0.2%) | 0 (0%) |
| M32.9 | SLE, unspecified | 4014 (94.2%) | 0 (0%) |
| N29.8 | Lupus nephritis | 134 (3.1%) | 0 (0%) |
| L93.0 | Discoid lupus erythematosus | 33 (0.8%) | 221 (25.3%) |
| L93.1 | Subacute cutaneous lupus erythematosus | 54 (1.3%) | 499 (57.2%) |
| L93.2 | Other local lupus erythematosus | 3 (0.1%) | 41 (4.7%) |
| M32.0 | Drug-induced SLE | 4 (0.1%) | 65 (7.4%) |
| R76.8 | Positive lupus anti-coagulant | 25 (0.6%) | 49 (5.6%) |

Note that some patients may have more than one diagnosis recorded.

**Supplementary Table 2. Total number of different medications received by patients during their recorded disease history**

|  | Total medication category: | | |
| --- | --- | --- | --- |
| Feature | Less than 3 drugs, n (%) | 3 – 5 drugs, n (%) | More than 5 drugs, n (%) |
| Total throughout full disease history | 2376 (68.2%) | 1108 (31.8%) | 61 (1.8%) |
| Disease duration: |  |  |  |
| 0 - 2.0 years | 1108 (80.1%) | 275 (19.9%) | 1 (0.1%) |
| 2.1 – 5.0 years | 563 (66.2%) | 272 (32%) | 15 (1.8%) |
| 5.1 – 10.0 years | 520 (57.5%) | 354 (39.2%) | 30 (3.3%) |
| Over 10 years | 185 (53.5%) | 146 (42.2%) | 15 (4.3%) |

**Supplementary Table 3.**

| Reason | Adverse condition | HCQ or CQ (n=2989) | MTX (n=986) | AZA (n=454) | MPA (n=400) | CYC (n=15) | CSA (n=18) | TAC (n=17) | IVIG (n=1) |
| --- | --- | --- | --- | --- | --- | --- | --- | --- | --- |
| Number of patients with reason for cessation recorded | - | 527 (17.6%) | 411 (41.7%) | 218 (48%) | 146 (36.5%) | 11 (73.3%) | 8 (44.4%) | 6 (35.3%) | 0 (0%) |
| Adverse Reaction, n (%) | - | 161 (5.4%) | 102 (10.3%) | 69 (15.2%) | 37 (9.2%) | 1 (6.7%) | 2 (11.1%) | 1 (5.9%) | 0 (0%) |
| Type of adverse event, n (%) | Rash (including maculopapular eruption due to drug) | 40 (4.8%) | 3 (2.9%) | 1 (1.4%) | 1 (2.9%) | 0 (0%) | 0 (0%) | 0 (0%) | 0 (0%) |
|  | Nausea, vomiting or diarrhoea | 33 (20.5%) | 28 (27.5%) | 33 (47.8%) | 14 (37.8%) | 0 (0%) | 0 (0%) | 1 (100%) | 0 (0%) |
|  | Retinal disorder (including hydroxychloroquine retinopathy, retinal disorder, eye review) | 19 (11.8%) | 0 (0%) | 0 (0%) | 0 (0%) | 0 (0%) | 0 (0%) | 0 (0%) | 0 (0%) |
|  | Macular degeneration | 3 (1.9%) | 0 (0%) | 0 (0%) | 0 (0%) | 0 (0%) | 0 (0%) | 0 (0%) | 0 (0%) |
|  | Headache | 4 (2.5%) | 2 (2%) | 3 (4.3%) | 2 (5.4%) | 0 (0%) | 0 (0%) | 0 (0%) | 0 (0%) |
|  | Malaise and fatigue | 0 (0%) | 5 (4.9%) | 0 (0%) | 1 (2.7%) | 0 (0%) | 1 (50%) | 0 (0%) | 0 (0%) |
|  | Neutropenia and drug-induced neutropenia | 0 (0%) | 7 (6.9%) | 5 (7.2%) | 1 (2.7%) | 0 (0%) | 0 (0%) | 0 (0%) | 0 (0%) |
|  | Abnormal liver function tests and elevated transaminases | 0 (0%) | 12 (11.85%) | 5 (7.2%) | 1 (2.7%) | 0 (0%) | 0 (0%) | 0 (0%) | 0 (0%) |
|  | Hepatotoxicity | 0 (0%) | 4 (3.9%) | 6 (8.7%) | 0 (0%) | 0 (0%) | 0 (0%) | 0 (0%) | 0 (0%) |
|  | Hair loss or alopecia | 3 (1.9%) | 6 (5.9%) | 1 (1.4%) | 0 (0%) | 0 (0%) | 0 (0%) | 0 (0%) | 0 (0%) |
|  | Mouth ulcers | 0 (0%) | 3 (2.9%) | 0 (0%) | 0 (0%) | 0 (0%) | 0 (0%) | 0 (0%) | 0 (0%) |
|  | Tinnitus | 7 (4.3%) | 0 (0%) | 0 (0%) | 0 (0%) | 0 (0%) | 0 (0%) | 0 (0%) | 0 (0%) |
